# Smartwatch-Based Maximum Oxygen Consumption Measurement for Predicting Acute Mountain Sickness: Diagnostic Accuracy Evaluation Study

**DOI:** 10.2196/43340

**Published:** 2023-07-06

**Authors:** Xiaowei Ye, Mengjia Sun, Shiyong Yu, Jie Yang, Zhen Liu, Hailin Lv, Boji Wu, Jingyu He, Xuhong Wang, Lan Huang

**Affiliations:** 1 Institute of Cardiovascular Diseases of People's Liberation Army The Second Affiliated Hospital Army Medical University (Third Military Medical University) Chongqing China

**Keywords:** VO2max, maximum oxygen consumption, smartwatch, cardiopulmonary exercise test, acute mountain sickness

## Abstract

**Background:**

Cardiorespiratory fitness plays an important role in coping with hypoxic stress at high altitudes. However, the association of cardiorespiratory fitness with the development of acute mountain sickness (AMS) has not yet been evaluated. Wearable technology devices provide a feasible assessment of cardiorespiratory fitness, which is quantifiable as maximum oxygen consumption (VO_2_max) and may contribute to AMS prediction.

**Objective:**

We aimed to determine the validity of VO_2_max estimated by the smartwatch test (SWT), which can be self-administered, in order to overcome the limitations of clinical VO_2_max measurements. We also aimed to evaluate the performance of a VO_2_max-SWT–based model in predicting susceptibility to AMS.

**Methods:**

Both SWT and cardiopulmonary exercise test (CPET) were performed for VO_2_max measurements in 46 healthy participants at low altitude (300 m) and in 41 of them at high altitude (3900 m). The characteristics of the red blood cells and hemoglobin levels in all the participants were analyzed by routine blood examination before the exercise tests. The Bland-Altman method was used for bias and precision assessment. Multivariate logistic regression was performed to analyze the correlation between AMS and the candidate variables. A receiver operating characteristic curve was used to evaluate the efficacy of VO_2_max in predicting AMS.

**Results:**

VO_2_max decreased after acute high altitude exposure, as measured by CPET (25.20 [SD 6.46] vs 30.17 [SD 5.01] at low altitude; *P*<.001) and SWT (26.17 [SD 6.71] vs 31.28 [SD 5.17] at low altitude; *P*<.001). Both at low and high altitudes, VO_2_max was slightly overestimated by SWT but had considerable accuracy as the mean absolute percentage error (<7%) and mean absolute error (<2 mL·kg^–1^·min^–1^), with a relatively small bias compared with VO_2_max-CPET. Twenty of the 46 participants developed AMS at 3900 m, and their VO_2_max was significantly lower than that of those without AMS (CPET: 27.80 [SD 4.55] vs 32.00 [SD 4.64], respectively; *P*=.004; SWT: 28.00 [IQR 25.25-32.00] vs 32.00 [IQR 30.00-37.00], respectively; *P*=.001). VO_2_max-CPET, VO_2_max-SWT, and red blood cell distribution width-coefficient of variation (RDW-CV) were found to be independent predictors of AMS. To increase the prediction accuracy, we used combination models. The combination of VO_2_max-SWT and RDW-CV showed the largest area under the curve for all parameters and models, which increased the area under the curve from 0.785 for VO_2_max-SWT alone to 0.839.

**Conclusions:**

Our study demonstrates that the smartwatch device can be a feasible approach for estimating VO_2_max. In both low and high altitudes, VO_2_max-SWT showed a systematic bias toward a calibration point, slightly overestimating the proper VO_2_max when investigated in healthy participants. The SWT-based VO_2_max at low altitude is an effective indicator of AMS and helps to better identify susceptible individuals following acute high-altitude exposure, particularly by combining the RDW-CV at low altitude.

**Trial Registration:**

Chinese Clinical Trial Registry ChiCTR2200059900; https://www.chictr.org.cn/showproj.html?proj=170253

## Introduction

In recent years, mountain climbing has become a popular activity for pleasure, work, and athletic competitions. However, inadequate acclimatization to hypobaric hypoxia results in a series of symptoms known as acute mountain sickness (AMS). AMS is relatively common among new travelers, affecting >30% of individuals ascending to 3500 m and >70% of those ascending above 6000 m [1]. AMS is characterized by the presence of headache in combination with other symptoms, including dizziness, fatigue, loss of appetite, and insomnia [2]. Although younger age, female gender, rapid ascent, low oxygen saturation (SpO_2_), and abnormal ventilatory response to exercise have been previously associated with AMS and its severity [3-5], susceptible individuals still need to be further identified, especially with more accuracy and practicality.

Maximum oxygen consumption (VO_2_max) is defined as the maximum capacity of the cardiovascular, respiratory, and muscular systems to deliver and utilize oxygen, which is reflected by an individual’s cardiorespiratory fitness [6-9]. VO_2_max is accurately measured by the cardiopulmonary exercise test (CPET) during a maximal graded exercise until exhaustion, which is considered the gold standard for cardiorespiratory functional assessment [10,11]. However, the use of direct measurements is limited, particularly at high altitude, as it is time-consuming and requires infrastructure and specialized personnel to conduct exercise assessments. Therefore, indirect measurement methods of VO_2_max (Firstbeat fitness test [FFT]) have been developed and have become advantageous due to the popularity of smart wearable devices [12,13]. Previous studies have reported that VO_2_max estimated by the FFT method is accurate and suitable for athletes owing to its lower exercise intensity [14]. However, for those who require to face the challenge of extreme high-altitude environments over a short period, the maximum intensity of exercise should also be avoided so as not to affect the acclimatization process. Previous studies have shown that the error of the FFT method is less than 5% at low altitudes [14]; however, it remains controversial whether it underestimates the true VO_2_max. Additionally, its performance at high altitudes has not been evaluated and compared with that of the gold standard.

VO_2_max decreases during acute or chronic exposure to high altitudes, which is mainly attributed to the reduction of PO_2_ [15,16]. Moreover, in terms of limiting VO_2_max, in addition to environmental factors, more attention is focused on the oxygen delivery pathway, central circulation [17], maximal cardiac output [18], oxygen-carrying capacity of the blood, ability to distribute that blood into the contracting muscles, and finally, the ability of the muscles to consume oxygen [19]. In other words, the above physiological processes and related indices involving oxygen transport may also be potential predictors of AMS [20]. Therefore, this study aims to compare the accuracy and consistency of VO_2_max obtained from CPET and smartwatch test (SWT) at different atmospheric pressures and to determine whether VO_2_max at low altitudes is correlated with AMS. Further, we tested the hypothesis that the combination of VO_2_max-SWT and red blood cell (RBC) distribution width-coefficient of variation (RDW-CV) may be more efficient in predicting AMS.

## Methods

### Participant Recruitment

We recruited 46 healthy adults (27 women and 19 men, age range 22-54 years) from Chongqing, China, based on the inclusion and exclusion criteria. All participants had lived at low altitudes (<500 m) for at least 10 years and had no recent history of high-altitude (>2500 m) exposure (in the last 6 months). Participants with any one of the following conditions were excluded: respiratory and cardiovascular diseases, malignant tumors, liver and kidney dysfunctions, and psychiatric disorders or neuroses that would not allow them to complete the questionnaires.

### Ethics Approval

The study protocol (ChiCTR2200059900) complied with the Declaration of Helsinki and was approved by the ethics committee of Xinqiao Hospital of Army Medical University (approval: 2022-研第-060-01). Written informed consent was obtained from all the participants after the study details, procedures, benefits, and risks were explained.

### Procedures

**Figure 1:**
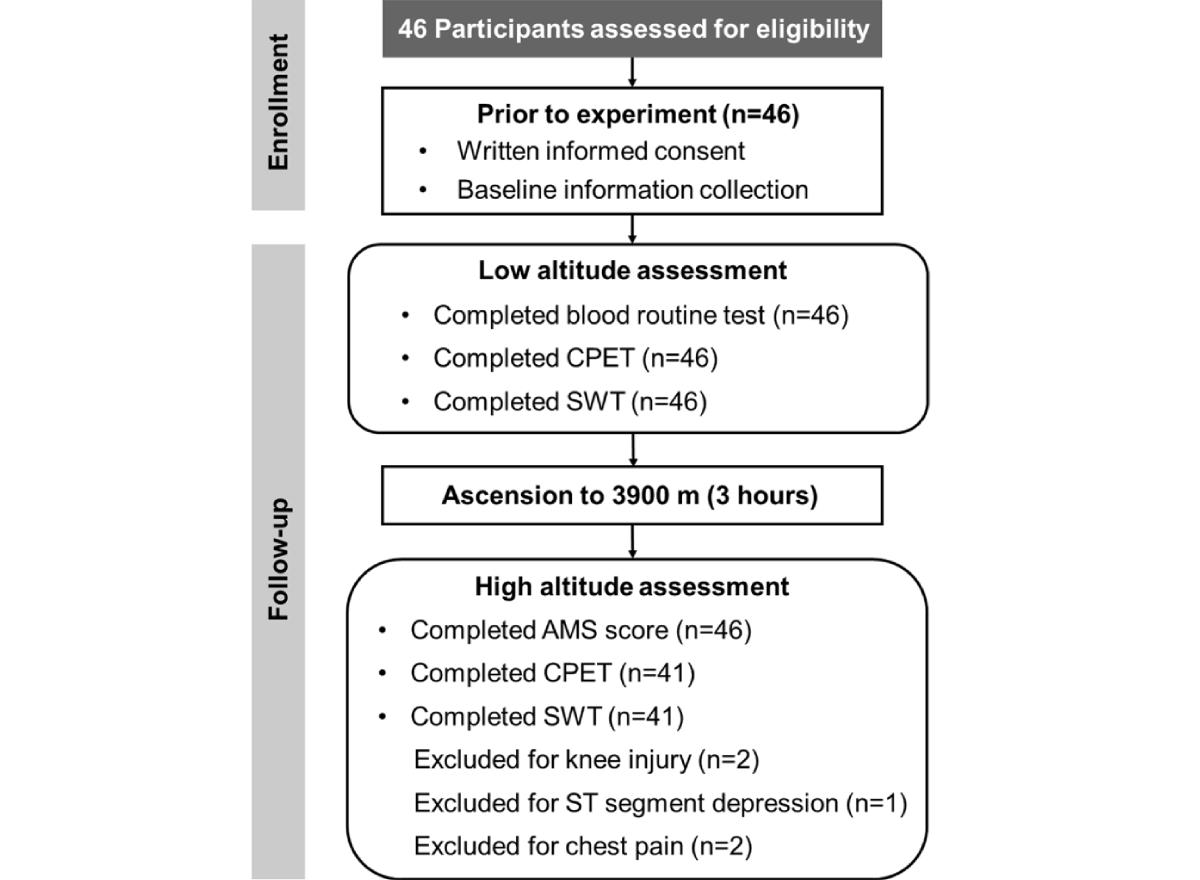
Cohort development diagram for this study. AMS, acute mountain sickness; CPET, cardiopulmonary exercise test; SWT, smartwatch test.

This study consisted of 2 exercise tests at low and high altitudes (Figure 1). The participants were instructed to avoid heavy load training 7 days before the tests and during the recovery days and to abstain from caffeine and alcohol for 24 hours before testing. On the first day of the study, each participant underwent a routine blood test before the SWT. After a 24-hour break, CPET was performed. The 2 tests (SWT and CPET) were performed at a similar time of day (SD 30 minutes) and were completed in 2 days. After resting for 3 days, the participants ascended to a high altitude (3900 m, Shigatse, China) in 3 hours by plane from a low altitude (300 m, Chongqing, China). On the second day at high altitude, they took 1 day off to complete the 2018 Lake Louise score assessment. Unfortunately, 2 individuals had knee injury because of the trip; therefore, they did not undergo exercise tests. Besides, 1 participant had an ST segment depression in the electrocardiogram and 2 participants had chest pain; therefore, they could not make it to the end. Finally, the remaining 41 participants repeated the exercise tests completely.

### Blood Routine Examination

The participants were required to avoid eating or drinking anything (fasting) apart from water for up to 12 hours. Approximately 5 mL of intravenous blood was collected from the inside of the elbow and mixed with 1 mL of dipotassium ethylenediaminetetraacetic acid anticoagulant by using a tight band (tourniquet). Blood samples were analyzed using a BC-3000 plus automated hematology corpuscle analyzer (Mindray). The details of the 19 different parameters are presented in Multimedia Appendix 1. Blood tests at low altitude were performed between 7 AM and 9 AM on the same day before the exercise tests. All biochemical parameters were measured in the blood samples at the Clinical Laboratory of Cardiology Science of Xinqiao Hospital, Army Medical University.

### CPET Analysis

The CPET was performed on an electronically braked cycle ergometer (EC3000e, Customed) in an erect position with breath-by-breath measurements through a tightly fitted face mask of minute ventilation, O_2_ uptake, and CO_2_ output by using a cardiopulmonary exercise testing system (Metalyzer 3B, Cortex). Before performing CPET, the baseline physiological measures for all devices used in this study were measured for 5 minutes in a resting state and subsequently in a standing position. After the baseline measurement, the test was conducted immediately. The cycle ergometry test protocol included 3 minutes of free-wheel cycling and subsequently proceeded with a continual increase in resistance by 25 W/min (according to the prior known exercise capacity [21], so that the test would last 10-12 minutes) until test completion or exhaustion. VO_2_max was defined as the highest 30-s average value within the last minute of exercise until the first 15 s of recovery at peak exercise [22]. Standard 12-lead electrocardiogram, blood pressure, and SpO_2_ were obtained at rest, every minute during exercise, and for ≥4 minutes during the recovery phase throughout the procedure using a 12-lead connection (custo-Cardio 3000BT-A, Cortex) in real time, blood pressure cuffs (Suntech Tango M2, Cortex) in the upper arm, and a finger clip portable oximeter (Nonin wristOx2), respectively.

### SWT Analysis

We provided participants with a smartwatch (Huawei Watch GT Runner) and instructed them to wear it correctly on the left wrist, which enables reliable and persistent measurement of running speed, distance, and heart rate. Therefore, these measurements could be monitored continuously and automatically during each running activity, stored on the participant’s mobile device (Huawei MatePad 11 DBY-W09), and regularly transmitted to a secure cloud server, which was later transferred to the Huawei Health Center software through Bluetooth. Specifically, VO_2_max estimation steps were as follows: (1) the personal background information (age, height, and weight) of the participant was logged in and the exercise type (running outdoors) was selected; (2) the participant started to run with a smartwatch that measured the heart rate and speed on level ground; (3) the start and end points were in the same place, and the smartwatch was stopped by the researchers uniformly with a timely click; (4) the researchers subsequently saved the participants’ running data to an album on the pad, facilitating further statistical analysis; and (5) the smartwatch and mobile device were formatted to prepare for the next test.

The signal processing by Huawei Watch GT Runner is licensed by the Firstbeat Technology’s Fitness Test, which is based on intelligent detection for both data reliability and exercise pattern during successive recording [13]. Briefly, the moving average filter was applied to both heart rate and physical activity data. After filtering the data, only data points at which both heart rate and physical activity data increased were selected as a period of physical activity. This was conducted by differentiating the data and selecting where both differentiated data were positive. The situations where the data series were excluded are listed below: (1) significant heart rate decreases and exceptional striding pattern (identified as a situation of running on a very steep downhill or soft surface automatically), (2) significant heart rate increases while the velocity remained 0 (identified as stopping suddenly in the middle), and (3) a short duration of highly increasing intensity (identified as insufficient effort level). After exclusions, the selected data series were further segmented as different heart rate zones according to the effort levels. Of them, the reliable data segments that belong to a long series of successive heartbeat intervals (in generally 20 s-10 minutes and preferably 30 s-4 minutes) and with a small heart rate change level were recognized as sufficient effort and used to calculate the VO_2_max. In these reliable segments, speed was measured based on acceleration measurements by using a satellite navigation system. VO_2_max estimates were made for each reliable segment by using the following theoretical VO_2_ equation: theoretical VO_2_ (mL·kg^–1^·min^–1^) = 3.5 * speed (km/h). The obtained VO_2_max for each data segment was weighed and subsequently utilized to make a linear equation for calculating the final VO_2_max (the detailed rule of weighting is shown in patent US9237868B2).

### Lake Louise Consensus Scoring System and AMS

The presence of AMS at high altitude was assessed using the Lake Louise consensus scoring system 2018 version [23]. According to the 4 main symptoms, namely, headache, gastrointestinal symptoms, fatigue/weakness, and dizziness/vertigo, the scores were 0, 1, 2, and 3 in the order of none, mild, moderate, and severe, respectively. A total score of ≥3 combined with headache can be diagnosed as AMS.

### Statistical Analyses

Categorical variables were described as numbers and percentages. Descriptive statistics were presented as mean (SD) for variables with skewed distribution and median (IQR) for variables with normal distribution. The Mann–Wilcoxon rank-sum, independent-sample *t* test (2-sided test), Pearson chi-square test, and Fisher exact tests were used to compare the continuous and categorical variables statistically. The correlation magnitude and coefficient of determination between VO_2_max-CPET and VO_2_max-SWT were assessed using Pearson correlation. The intraclass correlation coefficient and paired-sample *t* tests (2-sided test) were performed to determine the agreement between VO_2_max-CPET and VO_2_max-SWT at low and high altitudes, respectively. We calculated the mean absolute error and mean absolute percentage error (MAPE) to evaluate the accuracy of the estimation. Furthermore, we used a Bland–Altman plot to investigate the level of agreement with 95% limits of agreement [24].

The relationship between the variables and AMS was examined by binomial logistic regression analysis with univariate analyses. The relationship between VO_2_max-CPET, VO_2_max-SWT, RDW-CV, and AMS was further examined by multivariate analyses. In the preliminary screening, we considered the variable with *P*<.05 as a potential risk factor, and an adjusted binary logistic regression model subjected the variable to identify the independent risk factors for AMS after the adjustment. Receiver operating characteristic (ROC) curves were constructed, and the Youden index was calculated. The optimal cutoff of variables for diagnosing AMS was determined at the point where the Youden index was maximum on the ROC analysis. We also compared the ROC curves of VO_2_max-CPET, VO_2_max-SWT, and RDW-CV alone or in combination. Differences were considered statistically significant at *P*<.05. Statistical analyses were performed using the SPSS Statistics software (IBM Corp) for Windows (version 26) and MedCalc software for Windows.

## Results

### Participant Characteristics

A total of 46 participants were recruited for this study, of whom 20 (44%) participants developed AMS. The clinical characteristics of participants with AMS and without AMS are presented in Table 1. There were no differences in age, sex, BMI, baseline heart rate, SpO_2_, and blood pressure between the 2 groups. RBC count; hemoglobin, hematocrit, and mean corpuscular hemoglobin levels; mean corpuscular hemoglobin concentration; and RDW-SD did not differ significantly between the participants in the 2 groups, whereas the AMS group had higher RDW-CV at low altitude than the non-AMS group (14.25 [IQR 12.75-21.03] vs 12.70 [IQR 12.25-13.53], respectively; *P*=.02). In addition, the AMS group had lower VO_2_max both measured by CPET (27.80 [SD 4.55] vs 32.00 [SD 4.04], respectively; *P*=.004) and estimated by SWT (28.00 [SD 6.75] vs 32.00 [IQR 30.00-37.00], respectively; *P*=.001) than the non-AMS group.

**Table 1:** Baseline characteristics, blood routine test, and maximum oxygen consumption of the participants at low altitudes.

Variables			Total (n=46)	Participants with AMS^a^ (n=20)	Participants without AMS (n=26)	*P* value^b^
**Baseline characteristics**						
	Age (years), mean (SD)		33.33 (7.80)	33.85 (8.41)	32.92 (7.44)	.70
	**Gender, n (%)**					.17
		Female	27 (59)	14 (70)	13 (50)	
		Male	19 (41)	6 (30)	13 (50)	
	BMI (kg/m^2^), median (IQR)		22.19 (20.22-23.64)	21.89 (20.15-23.44)	22.40 (20.22-24.01)	.78
	**Alcohol use, n (%)**					.18
		Current drinker or ex-drinker	10 (22)	2 (10)	8 (31)	
		Never	36 (78)	18 (90)	18 (69)	
	**Smoking status, n (%)**					.92
		Current smoker or ex-smoker	6 (13)	2 (10)	4 (15)	
		Nonsmoker	40 (87)	18 (90)	22 (85)	
	HR^c^ (beats/min), mean (SD)		78.93 (9.69)	81.25 (9.72)	77.15 (9.46)	.16
	SpO_2_^d^ (%), median (IQR)		97 (96-98)	97 (96-98.75)	97 (96-98)	.72
	SBP^e^ (mm Hg), median (IQR)		112.00 (103.75-121.25)	112.00 (105.00-126.50)	112.00 (102.75-118.75)	.92
	DBP^f^ (mm Hg), mean (SD)		74.11 (11.11)	72.95 (14.24)	75.00 (8.12)	.54
**Blood routine test**						
	RBC^g^ (10^–9^/L), median (IQR)		4.51 (4.30-4.95)	4.40 (4.25-4.89)	4.71 (4.33-5.08)	.19
	HGB^h^ (g/L), mean (SD)		131.59 (10.03)	128.85 (11.45)	133.69 (8.42)	.11
	HCT^i^ (%), mean (SD)		44.10 (4.32)	43.31 (4.28)	44.70 (4.34)	.28
	MCV^j^ (fL), median (IQR)		94.10 (90.90-96.60)	94.35 (96.50-96.58)	94.00 (91.73-96.78)	.89
	MCH^k^ (pg), median (IQR)		28.77 (27.46-29.82)	28.54 (27.18-29.77)	28.82 (27.58-29.90)	.71
	MCHC^l^ (g/L), mean (SD)		304.62 (15.21)	304.46 (14.72)	304.75 (15.86)	.95
	RDW-CV^m^ (%), median (IQR)		13.10 (12.30-14.83)	14.25 (12.75-21.03)	12.70 (12.25-13.53)	.02
	RDW-SD^n^ (fL), median (IQR)		44.40 (41.48-46.68)	44.40 (41.00-46.83)	44.55 (41.48-46.70)	.84
**Cardiorespiratory fitness**						
	VO_2_max-CPET^o^ (mL·kg^–1^·min^–1^), mean (SD)		30.17 (5.01)	27.80 (4.55)	32.00 (4.64)	.004
	VO_2_max-SWT^p^ (mL·kg^–1^·min^–1^), median (IQR)		30.50 (27.75-34.25)	28.00 (25.25-32.00)	32.00 (30.00-37.00)	.001

^a^AMS: acute mountain sickness.

^b^Differences were considered statistically significant if *P*<.05.

^c^HR: heart rate.

^d^SpO_2_: oxygen saturation.

^e^SBP: systolic blood pressure.

^f^DBP: diastolic blood pressure.

^g^RBC: red blood cell.

^h^HGB: hemoglobin.

^i^HCT: hematocrit.

^j^MCV: mean corpuscular volume.

^k^MCH: mean corpuscular hemoglobin.

^l^MCHC: mean corpuscular hemoglobin concentration.

^m^RDW-CV: red blood cell distribution width-coefficient of variation.

^n^RDW-SD: red blood cell distribution width-standard deviation.

^o^VO_2_max-CPET: maximum oxygen consumption measured by cardiopulmonary exercise test.

^p^VO_2_max-SWT: maximum oxygen consumption estimated by smartwatch test.

### Accuracy and Consistency Analyses of VO2max Estimation in the SWT at Low and High Altitudes

**Figure 2:**
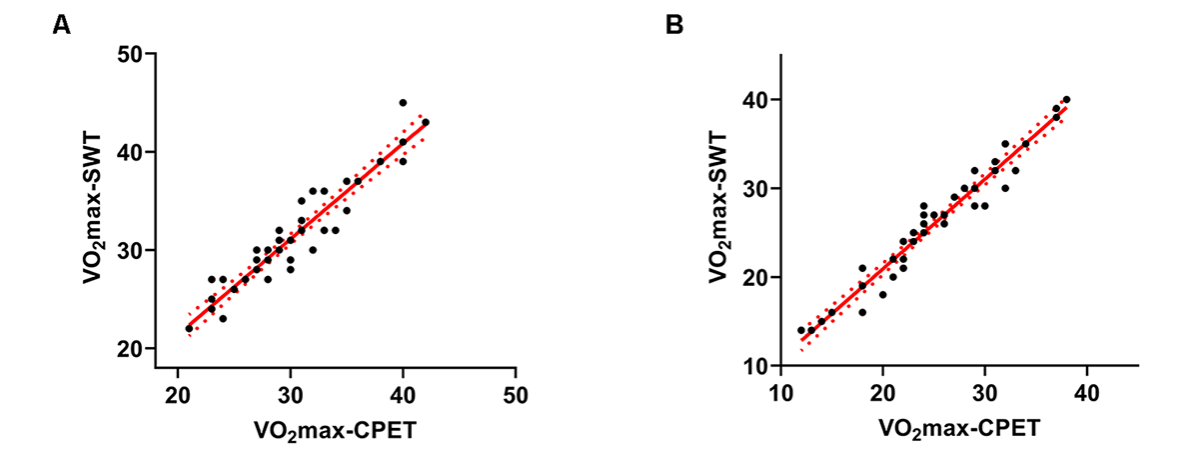
Linear regression plots between the estimated maximum oxygen consumption measured by smartwatch test and maximum oxygen consumption measured by cardiopulmonary exercise testing. Pearson correlation between the maximum oxygen consumption estimated by smartwatch and measured by cardiopulmonary exercise testing at low altitude (A) and at high altitude (B). The coefficient of determination (R2) and 95% CI bounds (dotted line) are depicted for the regression lines (solid). VO_2_max-CPET: maximum oxygen consumption measured by cardiopulmonary exercise testing; VO_2_max-SWT: estimated maximum oxygen consumption by smartwatch test.

**Figure 3:**
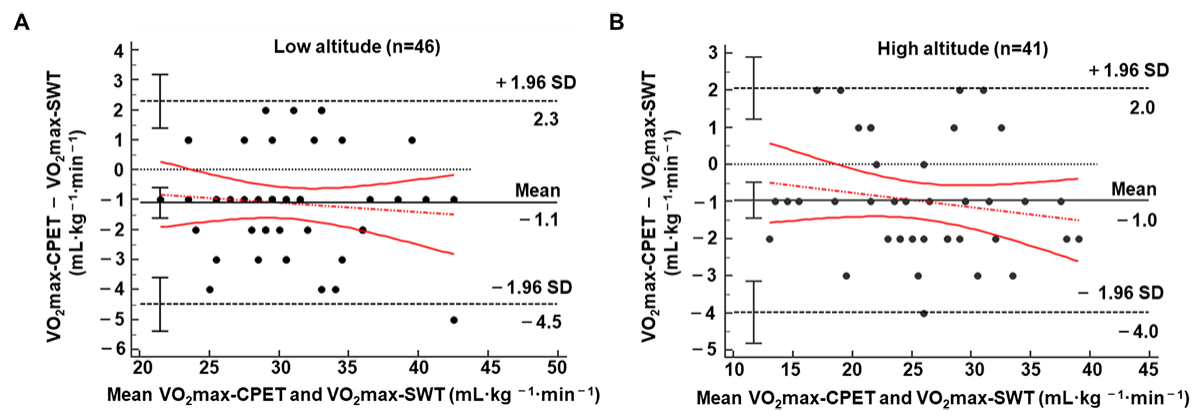
Bland-Altman plots between the estimated maximum oxygen consumption by smartwatch test and maximum oxygen consumption measured by cardiopulmonary exercise test at low altitude (A) and at high altitude (B). Mean biases (solid line), 95% limits of agreement (dashed line), and equality (dotted line) are also depicted. VO_2_max-CPET: maximum oxygen consumption measured by cardiopulmonary exercise test; VO_2_max-SWT: estimated maximum oxygen consumption by smartwatch test.

Table 2 shows the VO_2_max in the SWT and CPET at low and high altitudes. The values of VO_2_max-SWT were significantly overestimated at both low (constant error=1.11 [SD 1.73] mL·kg^–1^·min^–1^; *t*_45_=4.35; *P*<.001) and high (constant error=0.98 [SD 1.54] mL·kg^–1^·min^–1^; *t*_40_=4.05; *P*<.001) altitudes. A 6% MAPE (mean absolute error=1.761 mL·kg^–1^·min^–1^) at low altitude and a 6.8% MAPE (mean absolute error=1.610 mL·kg^–1^·min^–1^) at high altitude were observed, indicating a low average deviation between the 2 methods (Table 2). Furthermore, a strong correlation was found between VO_2_max-SWT and VO_2_max-CPET values (low altitude: *R*^2^=0.889; *P*<.001; high altitude: *R*^2^=0.947; *P*<.001; Figure 2). The results of the intraclass correlation coefficient revealed that VO_2_max-SWT had a good level of agreement with the directly measured VO_2_max-CPET at low (0.942; *P*<.001) and high (0.973; *P*<.001) altitudes. Additionally, the Bland–Altman plots demonstrated a small bias of the VO_2_max-SWT values compared to the VO_2_max-CPET at low (bias=1.11 mL·kg^–1^·min^–1^, Figure 3A) and high (bias=1.00 mL·kg^–1^·min^–1^, Figure 3B) altitudes. VO_2_max-SWT showed even a lower range of bias at high altitudes than at low altitudes (upper to lower limits of agreement: 6.0 mL·kg^–1^·min^–1^ vs 6.8 mL·kg^–1^·min^–1^, respectively).

**Table 2:** Correlations and differences between the estimated maximum oxygen consumption in the smartwatch test and the measured maximum oxygen consumption in the cardiopulmonary exercise test.

	VO_2_max-CPET^a^ (mL·kg^–1^·min^–1^), mean (SD)	VO_2_max-SWT^b^ (mL·kg^–1^·min^–1^), mean (SD)	CE^c^ (mL·kg^–1^·min^–1^), mean (SD)	*t* (df)	*r*	Intraclass correlation coefficient	Mean absolute error (mL·kg^–1^·min^–1^)	Mean absolute percentage error (%)
Low altitude (n=46)	30.17 (5.01)	31.28 (5.17)	1.11 (1.73)	4.35 (45) (*P*^d^<.001)	0.943 (*P*<.001)	0.942 (*P*<.001)	1.761	6
High altitude (n=41)	25.20 (6.46)	26.17 (6.71)	0.98 (1.54)	4.05 (40) (*P*<.001)	0.973 (*P*<.001)	0.973 (*P*<.001)	1.610	6.80

^a^VO_2_max-CPET: maximum oxygen consumption measured by the cardiopulmonary exercise test.

^b^VO_2_max-SWT: maximum oxygen consumption estimated by the smartwatch test.

^c^CE: constant error (arithmetic mean of the difference between estimated and measured VO_2_max).

^d^Differences were considered statistically significant if *P*<.05.

### Distribution of VO_2_max and Incidence of AMS

**Figure 4:**
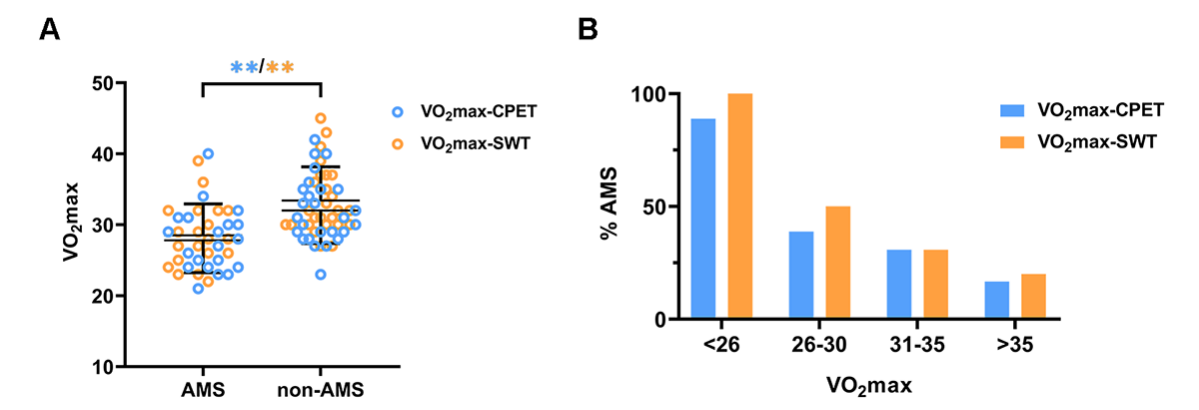
(A) Distribution of the estimated maximum oxygen consumption measured by smartwatch test and cardiopulmonary exercise test based on the diagnosis of acute mountain sickness. (B) Diagram of the probability of acute mountain sickness occurrence for different ranges of the maximum oxygen consumption value at low altitude (blue: maximum oxygen consumption measured by cardiopulmonary exercise test; orange: maximum oxygen consumption measured by smartwatch test). **Significantly different between acute mountain sickness and non–acute mountain sickness at *P*<.01. AMS: acute mountain sickness; VO_2_max-CPET: maximum oxygen consumption measured by cardiopulmonary exercise test; VO_2_max-SWT: estimated maximum oxygen consumption by smartwatch test.

There was a significant difference in the VO_2_max values at low altitude between the participants with and without AMS. VO_2_max-CPET in the AMS group was lower than that in the non-AMS group (27.80 [SD 4.55] vs 32.00 [SD 4.64], respectively; *P*=.004), and a similar result was revealed by SWT (28.00 [IQR 25.25-32.00] vs 2.00 [IQR 30.00-37.00], respectively; *P*=.001; Figure 4A). The distribution of the VO_2_max values based on the AMS results is shown in Figure 4B. Approximately 90% (16/17) of the participants with VO_2_max values <26 mL·kg^–1^·min^–1^ developed AMS compared with approximately 20% (3/15) of the participants who developed AMS with a VO_2_max>35 mL·kg^-1^·min^-1^. Patients with AMS seemed to have a lower VO_2_max, regardless of whether it was directly measured by CPET or estimated by SWT.

### Univariate and Multivariate Logistic Regression Analyses for AMS

To further explore the association between VO_2_max and AMS, a univariate analysis was performed. Table 3 shows that VO_2_max-CPET (odds ratio [OR] 0.807, 95% CI 0.686-0.949; *P*=.01) and VO_2_max-SWT (OR 0.765, 95% CI 0.635-0.922; *P*=.005) at low altitude and baseline RDW-CV (OR 1.177, 95% CI 0.999-1.386; *P*=.05) were potentially associated with AMS occurrence. Multivariate regression analysis identified VO_2_max-CPET (OR 0.770, 95% CI 0.640-0.926; *P*=.006) and RDW-CV (OR 1.263, 95% CI 1.028-1.553; *P*=.03) as well as VO_2_max-SWT (OR 0.720, 95% CI 0.578-0.898; *P*=.004) and RDW-CV (OR 1.273, 95% CI 1.027-1.577; *P*=.03) as independent factors associated with the development of AMS at high altitude.

**Table 3:** Binomial logistic regression analysis of factors related to acute mountain sickness.

Variables	Univariable		Multivariable		Multivariable	
	OR^a^ (95% CI)	*P* value^b^	OR (95% CI)	*P* value	OR (95% CI)	*P* value
Age (years)	1.016 (0.942-1.096)	.69	N/A^c^	N/A	N/A	N/A
Male (Y/N^d^)	2.333 (0.684-7.960)	.18	N/A	N/A	N/A	N/A
BMI (kg/m^2^)	1.037 (0.852-1.262)	.72	N/A	N/A	N/A	N/A
Tobacco (Y/N)	1.636 (0.268-9.980)	.59	N/A	N/A	N/A	N/A
Alcohol (Y/N)	4.000 (0.744-21.496)	.11	N/A	N/A	N/A	N/A
HR^e^ (beats/min)	1.048 (0.982-1.118)	.16	N/A	N/A	N/A	N/A
SpO_2_^f^ (%)	0.907 (0.558-1.472)	.69	N/A	N/A	N/A	N/A
SBP^g^ (mm Hg)	1.002 (0.965-1.040)	.91	N/A	N/A	N/A	N/A
DBP^h^ (mm Hg)	0.983 (0.932-1.037)	.53	N/A	N/A	N/A	N/A
VO_2_max-CPET^i^ (mL·kg^-1^·min^-1^)	0.807 (0.686-0.949)	.01	0.770 (0.640-0.926)	.006	N/A	N/A
VO_2_max-SWT^j^ (mL·kg^–1^·min^–1^)	0.765 (0.635-0.922)	.005	N/A	N/A	0.720 (0.578-0.898)	.004
RBC^k^ (10^–9^/L)	0.711 (0.219-2.308)	.57	N/A	N/A	N/A	N/A
HGB^l^ (g/L)	0.949 (0.889-1.012)	.11	N/A	N/A	N/A	N/A
HCT^m^ (%)	0.923 (0.800-1.066)	.28	N/A	N/A	N/A	N/A
MCV^n^ (fL)	0.966 (0.876-1.064)	.48	N/A	N/A	N/A	N/A
MCH^o^ (pg)	0.892 (0.660-1.205)	.46	N/A	N/A	N/A	N/A
MCHC^p^ (g/L)	0.999 (0.961-1.038)	.95	N/A	N/A	N/A	N/A
RDW-CV^q^ (%)	1.177 (0.999-1.386)	.05	1.263 (1.028-1.553)	.03	1.273 (1.027-1.577)	.03
RDW-SD^r^ (fL)	0.985 (0.850-1.141)	.84	N/A	N/A	N/A	N/A

^a^OR: odds ratio.

^b^Differences were considered statistically significant if *P*<.05.

^c^N/A: not applicable.

^d^Y/N: yes/no.

^e^HR: heart rate.

^f^SpO_2_: oxygen saturation.

^g^SBP: systolic blood pressure.

^h^DBP: diastolic blood pressure.

^i^VO_2_max-CPET: maximum oxygen consumption measured by cardiopulmonary exercise test.

^j^VO_2_max-SWT: maximum oxygen consumption estimated by smartwatch test.

^k^RBC: red blood cell.

^l^HGB: hemoglobin.

^m^HCT: hematocrit.

^n^MCV: mean corpuscular volume.

^o^MCH: mean corpuscular hemoglobin.

^p^MCHC: mean corpuscular hemoglobin concentration.

^q^RDW-CV: red blood cell distribution width-coefficient of variation.

^r^RDW-SD: red blood cell distribution width-standard deviation.

### VO_2_max-Based Model for Predicting AMS

**Figure 5:**
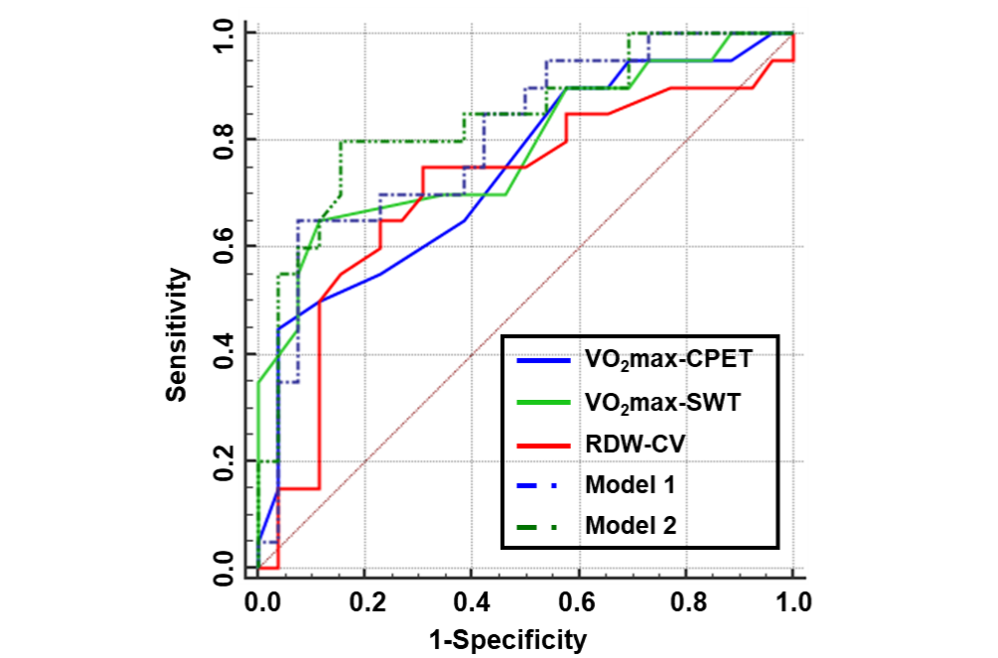
Receiver operating characteristic curves for maximum oxygen consumption measured by the cardiopulmonary exercise test (blue solid line), estimated maximum oxygen consumption by the smartwatch test (green solid line), and red blood cell distribution width-coefficient of variation (red solid line), and for Model 1 (blue dotted line: combination of maximum oxygen consumption measured by cardiopulmonary exercise test and red blood cell distribution width-coefficient of variation) and Model 2 (green dotted line: combination of estimated maximum oxygen consumption by smartwatch test and red blood cell distribution width-coefficient of variation) in predicting acute mountain sickness. VO_2_max-CPET: maximum oxygen consumption measured by cardiopulmonary exercise test; VO_2_max-SWT: estimated maximum oxygen consumption by smartwatch test; RDW-CV: red blood cell distribution width-coefficient of variation.

As both VO_2_max and RDW-CV were closely related to AMS, we constructed the combined predictive models for AMS. Table 4 and Figure 5 show the area under the curve (AUC) of VO_2_max-CPET (AUC 0.743, 95% CI 0.597-0.889), VO_2_max-SWT (AUC 0.785, 95% CI 0.646-0.923), and RDW-CV (AUC 0.708, 95% CI 0.547-0.868).

Either the AUC of the VO_2_max-CPET or VO_2_max-SWT was higher than that of RDW-CV (both *P*>.05, Table 4). For the VO_2_max-SWT, a sensitivity of 65% and a specificity of 88.46% were observed at the optimal cutoff value of 29.5 mL·kg^–1^·min^–1^, with a higher positive predictive value of 81.25% and a negative predictive value of 76.67%. However, no significant difference was found in AUC when compared to the VO_2_max-CPET (0.785 vs 0.743, respectively; *P*=.25). Although the independent indicators were effective and significant, this combined predictive model was more accurate when VO_2_max and RDW-CV were combined. In other words, the combined model 2 enhanced the diagnostic power with modest AUC gains of 0.03-0.06, although not statistically different from model 1 (0.839 vs 0.804, respectively; *P*=.27) or VO_2_max-SWT alone (0.839 vs 0.785, respectively; *P*=.28). The combined model 2 also improved the prediction of AMS by increasing sensitivity from 65% to 80% compared with VO_2_max-SWT alone while attaining high specificity.

**Table 4:** Receiver operating characteristic curves to assess the performance of the maximum oxygen consumption measured by cardiopulmonary exercise test and the maximum oxygen consumption estimated by smartwatch test in predicting acute mountain sickness.^a^

	AUC^b^ (95% CI)	Optimal cutoff values (mL·kg^–1^·min^–1^ or fL)	Sensitivity (%)	Specificity (%)	PPV^c^ (%)	NPV^d^ (%)
VO_2_max-CPET^e^	0.743 (0.597-0.889)	26.50	45	96.15	90	69.44
VO_2_max-SWT^f^	0.785 (0.646-0.923)	29.50	65	88.46	81.25	76.67
RDW-CV^g^	0.708 (0.547-0.868)	13.10	75	69.23	65.22	78.26
Model 1: VO_2_max-CPET + RDW-CV	0.804 (0.675-0.933)	N/A^h^	65	92.31	87.50	77.42
Model 2: VO_2_max-SWT + RDW-CV	0.839 (0.720-0.959)	N/A	80	84.62	80	84.62

^a^Comparison of area under the curve: maximum oxygen consumption measured by cardiopulmonary exercise test versus maximum oxygen consumption estimated by smartwatch test (*P*=.25); Model 1 versus maximum oxygen consumption measured by cardiopulmonary exercise test (*P*=.22); Model 2 versus maximum oxygen consumption estimated by smartwatch test (*P*=.28); Model 1 versus Model 2 (*P*=.27).

^b^AUC: area under the curve.

^c^PPV: positive predictive value.

^d^NPV: negative predictive value.

^e^VO_2_max-CPET: maximum oxygen consumption measured by cardiopulmonary exercise test.

^f^VO_2_max-SWT: maximum oxygen consumption estimated by smartwatch test.

^g^RDW-CV: red blood cell distribution width-coefficient of variation.

^h^N/A: not applicable.

## Discussion

### Principal Results

Our study comparatively evaluated the VO_2_max (CPET vs SWT) of individuals at a low altitude and subsequently at a high altitude. We demonstrated that the smartwatch device was a feasible and accurate tool for assessing cardiorespiratory fitness at both altitudes. We also proposed a novel model based on smartwatch-derived VO_2_max with good performance in predicting AMS. Our easy-to-use approach for estimating VO_2_max can be more widely applied for screening individuals susceptible to AMS on a large scale.

Previous clinical trials [25-27] have shown that VO_2_max ranged from 20 mL·kg^–1^·min^–1^ to 50 mL·kg^–1^·min^–1^ according to the sex, age, or ethnicity of participants. In our study, the value of VO_2_max measured either in CPET (low altitude: 30.17 [SD 5.01] vs high altitude: 25.20 [SD 6.46]; *P*<.001) or SWT (low altitude: 31.28 [SD 5.17] vs high altitude: 26.17 [SD 6.71]; *P*<.001) was within a fair level range, reflecting the sedentary fitness of this cohort. The 10 pairs of participants’ VO_2_max at low altitude were nearly the same. The high overlap rate of our data may be because the measurement output from CPET and SWT are integers, which reduce the numerical difference. In other words, we consider that when the difference between the 2 measurements is less than 1, this difference may not show up. Such a high overlap rate outcome has been reported in a previous study [12]. Besides, repeated measures may help reduce the repetitive rate.

Similar to that shown in a previous report, VO_2_max was lower by approximately 16.5% at 3900 m evaluated by both CPET and SWT compared to that at low altitude in our trial, which can be attributed to the reduction of atmospheric PO_2_ at high altitude [28]. Interestingly, the VO_2_max-SWT was slightly higher than the VO_2_max measured by the breath-to-breath method at both low and high altitudes. Therefore, we considered that the following issues may be associated with the differences: (1) the exercise mode in SWT was accelerative running, which requires a larger number of muscle groups, whereas the mode of CPET was a cycle ergometer, mostly relying on endurance, and (2) CPET required maximal performance; however, SWT only required submaximal exercise intensity. In addition, the overestimated VO_2_max of the SWT was more evident at high altitude, as highlighted by the higher MAPE (6.80% vs 6%, respectively) and wider 95% limits of agreement criterion. Interestingly, an increase in altitude did not significantly affect the *R*^2^ and intraclass correlation coefficient values of VO_2_max assessed by the SWT, suggesting its high compatibility for hypobaric hypoxic conditions. To keep up with the exercise pattern used in the SWT, it is more rigorous to perform the CPET program on a treadmill than on a cycle ergometer to minimize the impact of the different muscular factors. However, there are several distinct differences between a treadmill and a bicycle ergometer (ie, space, safety, and costs)—all of which are conducive to a bicycle ergometer. It is only possible to change the slope and speed in the treadmill exercise. However, a cycle ergometer attains the linearity in workload increment well [29]. Thus, a cycle ergometer program should be preferred over a treadmill program at high altitudes. Hence, we finally opted to use a cycle ergometer for CPET. Nevertheless, the VO_2_max measured by the 2 methods has good correlation and consistency.

VO_2_max evaluation using CPET is inconvenient in practice. In the past few decades, several new methods for estimating VO_2_max have been investigated through a submaximal exercise protocol, including the Queen college step test [30], 20-m shuttle run test [31], and PWC170 [32,33]. Although they are easy to perform, the accuracy of the indirect method in estimating VO_2_max remains controversial. Thus, more variables such as basic parameters (age, sex, BMI) [34] and exercise indicators (maximal heart rate, speed, and covered distance) [35,36] were utilized in discrepant equations for more accuracy in subsequent studies. For instance, Marsh [37] found a 4-stage incremental running program estimating VO_2_max well (standard error of estimate=3.98-4.08 mL·kg^–1^·min^–1^; *r*=0.642-0.646). However, this equation cannot be applied to the general population because correlation data were obtained for male athletes [37]. Instead of simply substituting variables into the equation, the smartwatch employed an algorithm called *Firstbeat* to evaluate VO_2_max in daily life. One of the key features of this patented technology is monitoring the running speed along with the heart rate continuously during each workout and automatically excluding the data without a linear relationship. A white paper of *Firstbeat* claimed that based on a database of 2690 freely performed runs by 79 individuals, its accuracy was up to 95% (MAPE<5%) and the error was below 3.5 mL·kg^–1^·min^–1^ [14]. For perspective, it is superior to most other indirect submaximal tests (10%-15%) and approaches the direct laboratory test (approximately 5%). Thus, the FFT method can be commonly used to estimate VO_2_max when high-intensity exercise is limited or laboratory equipment is unavailable. Düking et al [38] found a coefficient of variation of 4% between Garmin watch and the criterion measure over the VO_2_ peak range from 38 mL·kg^–1^·min^–1^ to 61 mL∙ kg^–1^·min^–1^ through a small sample study. In this regard, the smartwatch with FFT is a portable device with satisfactory accuracy for estimating VO_2_max in the submaximal exercise protocol. Importantly, they also reported that the MAPE between the smartwatch and criterion measure was 7.1% when analyzing VO_2_ peak below 45 mL·kg^–1^·min^–1^ whose discrepancy was less inaccurate in our study. The MAPE in this study was 6% and 6.8% at low and high altitudes, respectively. In [18], the 23 participants were of Caucasian origin, while in our study, the 46 participants were Chinese healthy adults. Hence, different race, gender, age, height, body weight and physical activity level of individuals may well explain differences in VO_2_max, both between the other reports [39] and this study.

VO_2_max represents the maximum oxygen utilization capacity of an individual. In normoxic conditions, a higher VO_2_max indicates greater exercise capacity and better cardiorespiratory fitness [40-42]. However, due to PO_2_ decrease (approximately 63% of low altitude at 3700 m) at high altitude, arterial SpO_2_ and the amount of oxygen that eventually reaches the tissue and organ are reduced [43]. Thus, aerobic metabolic capacity and VO_2_max are significantly inhibited. To cope with hypoxia at high altitude, the body undergoes a series of physiological compensations in the cardiovascular and respiratory systems, such as increased heart rate [44,45], blood pressure [46], and respiratory rate [47]*.* Although these compensatory responses can compensate for oxygen insufficiency in a short period, long-term exposure may lead to irreversible changes such as pulmonary hypertension, chronic pulmonary disease, and heart failure [48,49]. VO_2_max has been an effective predictive indicator of mortality and rehospitalization in patients with chronic cardiovascular and respiratory diseases [50-52]. However, it has rarely been reported that VO_2_max contributes to the prognosis and rehabilitation of acute and chronic mountain illnesses [50]. In this study, we present the first evidence of VO_2_max as a predictor of AMS in a clinical trial.

It can be concluded from our results that individuals with a higher VO_2_max are unlikely to develop AMS. ROC analysis demonstrated that VO_2_max-CPET and VO_2_max-SWT showed a similar predictive value, particularly for VO_2_max-SWT, with a specificity up to 88.46% for a cutoff value of 29.5 mL·kg^–1^·min^–1^. The high specificity ensures a low incidence of AMS when VO_2_max is below the cutoff value of lowlanders unsuitable for acute high-altitude exposure. In addition, we found that RDW-CV was more closely related to AMS than other routine blood parameters. We believe that the low RDW-CV represents a uniform distribution of erythrocyte developmental states, indicating a more effective compensation response of RBC under acute hypoxia stress [53,54]. Although RDW-CV was an independent predictor for AMS and RDW-CV combined with VO_2_max showed a higher AUC, it is more convenient for individuals to obtain information on the potential suffering probability by using a smartwatch in daily life. Moreover, models 1 and 2 in Table 4 showed no statistical difference in AMS prediction compared to the single VO_2_max model. Therefore, SWT-based VO_2_max estimation can conveniently identify AMS-susceptible individuals and help evaluate the cardiorespiratory function and working capacity, which can benefit high-altitude travelers and workers and reduce the consumption of medical resources at high altitude. Although we cannot extend our validity claims to the entire population due to the small sample size and insufficiently diverse population characteristics, the conclusion that people with lower oxygen intake are more likely to develop AMS is well-founded and has been recently reported [55].

The VO_2_max estimated by SWT and measured by CPET has high consistency, indicating that smartwatches may replace the CPET system to obtain VO_2_max accurately and objectively by monitoring the common physical activities with a portable, low-cost system. After each exercise, the smartwatch can measure and record the exercise information, integrate the calculation, and update the VO_2_max value. In addition, VO_2_max measured at low altitudes is highly correlated with the occurrence of AMS, with satisfactory prediction performance. Therefore, it is feasible to use a smartwatch to measure VO_2_max at low altitudes to evaluate the possibility of AMS. In the future, this will benefit tourists, temporary workers, and other individuals who plan to travel at high altitudes and will help in identifying participants susceptible to AMS before high-altitude exposure.

To advance this field, several measures are required. First, there is an urgent need for validation standards for smartwatch devices to enable standardized research. Second, the open disclosure of commercial validation studies can enable better resource usage, as studies will not have to be repeated unnecessarily. Third, further development of smartwatch devices will allow new possibilities in the field of VO_2_max monitoring. Finally, subsequent trials should continue to focus on validating these devices compared to conventional standards and broaden their use and demonstrate new possibilities for accurate VO_2_max monitoring.

### Limitations

This study had some limitations. Notably, the Lake Louise consensus scoring system (2018 version) is subjective; therefore, we described each symptom as clearly as possible and provided necessary instructions before the participants completed the questionnaire to deal with the subjectivity. Second, running exercise instead of cycling should be implemented by CPET to minimize the inconsistencies in VO_2_max with SWT, which was not applied here due to the great safety risk at high altitude. Further studies and repeated measures are required to develop and investigate the predictive models of the SWT method based on submaximal running programs in terms of validity and reliability [11,56]. The present predictive models of the SWT method cannot be extended to the entire population. In the long term, conducting long-term validation research within a large and representative population is the scope of future studies, as the smartwatch will be extensively used by people. In addition, this study has insufficient reliability, as repeated measures analysis was not performed. It is contradictory to perform repeated measurements because measuring on the same day is limited by physical strength. Repeated measurements on different days also affect the assessment of AMS.

### Conclusions

Our findings demonstrate that VO_2_max estimated by SWT and CPET have good accuracy and agreement at both low and high altitudes. Importantly, smartwatch-based VO_2_max at low altitudes was a convenient and effective approach to predict AMS and to identify susceptible individuals following acute high-altitude exposure, particularly by combining the RDW-CV at low altitudes.

## Appendix

Multimedia Appendix 1 Original data of the cardiopulmonary exercise test and smartwatch test in this study.

## Abbreviations

AMS: acute mountain sickness
AUC: area under the curve
CPET: cardiopulmonary exercise test
FFT: Firstbeat fitness test
MAPE: mean absolute percentage error
OR: odds ratio
RBC: red blood cell
RDW-CV: red blood cell distribution width-coefficient of variation
ROC: receiver operating characteristic
SpO2: oxygen saturation
SWT: smartwatch test
VO_2_max: maximum oxygen consumption

## Data Availability

The data sets generated and analyzed during this study are available from the corresponding author on reasonable request.
